# Management of sudden cardiac death in cardiac sarcoidosis using the wearable cardioverter defibrillator

**DOI:** 10.1371/journal.pone.0194496

**Published:** 2018-03-22

**Authors:** Dirk Skowasch, Steven Ringquist, Georg Nickenig, René Andrié

**Affiliations:** 1 Department of Internal Medicine II, Division of Cardiology and Pneumology, University of Bonn, Bonn, Germany; 2 ZOLL, Pittsburgh, Pennsylvania, United States of America; Klinikum Region Hannover GmbH, GERMANY

## Abstract

**Background:**

Patients with cardiac sarcoidosis are at increased risk of ventricular tachycardia/fibrillation.

**Objective:**

We tested the hypothesis that the wearable cardioverter defibrillator can be used to mitigate the risk of sudden cardiac death among cardiac sarcoidosis patients.

**Methods:**

A retrospective review of the commercial database identified cardiac sarcoidosis patients who wore the wearable cardioverter defibrillator. Evidence for cardiac sarcoidosis diagnosis as well as demographic, co-morbidity and left ventricular ejection fraction were provided by patient clinical records. Clinical data also included daily wearable cardioverter defibrillator wear, shock treatment and survival information.

**Results:**

The wearable cardioverter defibrillator was worn by 46 cardiac sarcoidosis patients, 24 (52%) male. The median age was 48 years and median left ventricular ejection fraction was 30%. The wearable cardioverter defibrillator was worn a median of 23.6 hours each day. There were 11 ventricular tachycardia/fibrillation episodes occurring in 10 (22%) patients. Ventricular tachycardia/fibrillation occurred over a range of 1 to 79 days, median 24 days. First-shock success for conversion of ventricular tachycardia/fibrillation was 100%. Patient survival 24 hours after shock treatment was 100%. Follow up to determine the reason for discontinuing wearable cardioverter defibrillator use indicated that among shocked patients 7 received an implantable cardioverter defibrillator, 1 patient was admitted to the hospital ending in death 2 weeks after discontinuing wearable cardioverter defibrillator use, and 2 patients were lost to follow up. Among the not shocked patients, there were 16 who received an implantable cardioverter defibrillator while 7 achieved improved left ventricular ejection fraction.

**Conclusion:**

Management of sudden cardiac death among cardiac sarcoidosis patients was aided by the wearable cardioverter defibrillator resulting in successful termination of ventricular tachycardia/fibrillation upon delivery of shock.

## Introduction

Sarcoidosis is a systemic non-caseating granulomatous disorder of unknown etiology which affects the respiratory system in the majority of the cases; other organs including skin, eyes, nerves, liver and heart may also be afflicted. The etiology remains unknown, however evidence suggests that it is the product of an endogenous genetic susceptibility and an unknown antigenic stimulus [1,2]. Among sarcoidosis patients, cardiac sarcoidosis (CS) is reported clinically in 5% of patients, but an autopsy study as well as cardiovascular magnetic resonance (MRI) studies revealed that subclinical CS is found in about 25% of patients of [Western] European descent and even higher in Japanese patients [3–10].

While the general course of sarcoidosis is short and favorable, the prognosis is worse when cardiac manifestations exist, as this increases the risk of conduction abnormalities and heart failure. Among CS patients, the occurrence of ventricular tachycardia/fibrillation (VT/VF) is elevated, as evidenced by an event rate of 7.1% per year of appropriate implantable cardioverter defibrillator (ICD) therapies [11]. A recently published case study involving a young CS patient has described use of the WCD to manage VT/VF and prevent sudden cardiac death (SCD) [12]. There are, however, few data to help with SCD risk stratification [13]. A current consensus statement suggested that in a primary prevention group, the left ventricular ejection fraction (LVEF) should be reassessed after heart failure medication optimization and immunosuppression if indicated [6].

There is substantial literature supporting use of the WCD to manage individuals at increased risk for SCD due to VT/VF, such as after myocardial infarction or newly diagnosed heart failure when an ICD decision is not yet appropriate, or when the risk of VT/VF is due to transient or correctable causes [14]. Other patients identified as benefiting from the WCD include patients with a contraindication for ICD implantation: patients after ICD explantation undergoing prolonged antibiotic therapy; patients with a high risk of SCD but without a definitive diagnosis; end stage or transplant listed heart failure patients; and patients at increased but temporary risk for arrhythmia, such as those diagnosed with transient myocarditis [14–16].

Efficacy of the WCD has been evaluated in several publications. In two US based studies involving 3,569 and 8,453 subjects the WCD converted 99% and 91% of VT/VF events, respectively [17,18]. Recently a cohort from Germany involving 6,043 subjects, and representing the first analysis of a large cohort outside of the US healthcare system, also showed the WCD to be efficacious converting 94% of VT/VF events [19].

The current study was performed in order to examine how the WCD has been used in the management VT/VF in CS patients. The study utilized WCD wear data along with available clinical records to examine the characteristics and outcomes of 46 patients who wore the WCD.

## Materials and methods

### Consent

All patients consented to have their data used for quality monitoring, health care operation activities and research purposes.

### Patient cohort

Included were patients who were prescribed and wore the WCD from January, 2005 to June, 2014, and in whom the prescribing physician indicated the diagnosis of CS and an ICD 9 code of 135 (sarcoidosis) or 425.4 (other restrictive cardiomyopathy). Anonymized clinical records consisting of the patient’s history and physical examination available during dispensation of the WCD provided primary evidence for CS, demographic data, the presence of co-morbidities and LVEF.

### WCD use

The commercial database provided WCD use data to evaluate patient days of wear and hours of daily use. A day of wear was defined as any day on which the WCD was worn >15 minutes. Hours of daily use was defined as the hours of wear divided by 24 hours per day. On the first day of wear, however, hours of daily use was defined as the ratio of hours of WCD wear and the number of hours remaining that day, normalized to 24 hours. On the last day of wear, due to discontinuation of the WCD, hours of daily use was not determined.

### WCD treatment events

The WCD records the automated detection and shock treatment of sustained VT and VF arrhythmia events. A single event was defined to include all recordings of sustained VT or VF arrhythmias occurring within 24 hours of the index arrhythmia. Sustained VT or VF were ventricular tachyarrhythmia lasting longer than 30 seconds, with VT defined as having a consistent morphology and VF defined as an inconsistent morphology. ECG recordings with VT leading to VF were reported as VT/VF.

Data associated with shock events include the electrocardiogram (ECG) tracing encompassing the event, the number of shock treatments administered and whether shock resulted in conversion. Survival 24 hours post shock was determined by phone follow up with shock treated individuals, their families, or responsible medical professionals having direct knowledge of the event. Interpretation of patient data, including analysis of ECG tracings, was performed by the authors.

### Statistical analysis

Data are reported as the median and interquartile range (IQR) or as the number of events and the percentage of total events. Data analyses were performed using R version 3.0.3. Evaluation of data was performed using the Wilcoxon rank sum test for continuous data and Fisher’s exact test for categorical data. Statistical significance was defined as p-value <0.05.

## Results

### Patient profile and clinical characteristics

CS patients (N = 46) who were prescribed and wore the WCD in order to manage risk of VT/VF were identified. Table 1 summarizes the patient population by age, gender, associated co-morbidity and LVEF. There were 10 (22%) patients who were treated for VT/VF while wearing the WCD. Comparison of patients who were shocked versus those not shocked revealed similar distributions of gender and age. Patients presented co-morbidities commonly associated with CS. For example, congestive heart failure was reported in 22 (48%) patients and history of ventricular arrhythmia, syncope, heart block and coronary artery disease were also noted. LVEF measurement at the start of WCD use averaged 30%. In contrast to the other features recorded at the beginning of WCD use, the starting LVEF differed when treated and not treated patients were compared with values of 21% and 48%, respectively (Table 1). Additional raw data underlying the study are given in S1 Table.

**Table 1:** Baseline characteristics of cardiac sarcoidosis patients.

	All Patients (N = 46)	Shocked Patients (N = 10)	Not Shocked Patients (N = 36)	p-value
*Demographic*				
Male, N (%)	24 (52%)	6 (60%)	18 (50%)	0.73
Age, median [IQR]	48 [41–55]	47 [41–52]	48 [42–56]	0.79
*Co-Morbidity*, *N (%)*				
Congestive Heart Failure	22 (48%)	7 (70%)	15 (42%)	0.16
Ventricular Arrhythmia	19 (41%)	4 (40%)	15 (42%)	1
Syncope	15 (33%)	2 (20%)	13 (36%)	0.46
Heart Block	8 (17%)	1 (10%)	7 (19%)	0.66
Coronary Artery Disease	5 (11%)	2 (20%)	3 (8%)	0.30
*LVEF*, *median [IQR]*				
LVEF (%)	30 [21–58]	21 [20–29]	48 [25–60]	7.0x10^-3^

Abbreviations: Interquartile Range, IQR; Left Ventricular Ejection Fraction, LVEF; Number, N.

#### WCD use

Table 2 summarizes use of the WCD by days of wear and hours of daily use along with the reason for discontinuation of the WCD. No significant difference was observed when comparing shocked and not shocked patients. Among patients shocked in response to VT/VF the median length of wear was 42 days and median daily use was 23.8 hours, roughly 99% of the hours available per day. For not shocked patients, length of wear and daily use were 33 days and 23.3 hours, respectively. The most common reason given for WCD end of use was ICD implantation for 7 (70%) and 16 (44%) of the shocked and not shocked patients, respectively. Improvement of LVEF was recorded for 7 (19%) of the not shocked patients. For the overall CS patient population, there were 3 (7%) patients deaths, however, no death occurred during WCD use.

**Table 2:** Wearable cardioverter defibrillator experience.

	All Patients (N = 46)	Shocked Patients (N = 10)	Not Shocked Patients (N = 36)	p-value
*Wear Summary*				
Total Days of Wear, median [IQR]	33 [23–66]	42 [14–66]	33 [23–64]	0.81
Daily Use (Hours), median [IQR]	23.6 [22.4–23.9]	23.8 [23.6–23.9]	23.3 [21.4–23.9]	0.09
*End of Use Reason*, *N (%)*				
Received ICD	23 (50%)	7 (70%)	16 (44%)	0.28
LVEF Improved	7 (15%)	0 (0%)	7 (19%)	0.32
Deceased	3 (7%)	1 (10%)	2 (6%)	0.53
Other Non-Cardiac	13 (28%)	2 (20%)	11 (31%)	0.70

Abbreviations: Implantable Cardioverter Defibrillator, ICD; Interquartile Range, IQR; Left Ventricular Ejection Fraction, LVEF; Number, N.

### Shock treatment summary

**Fig 1:**
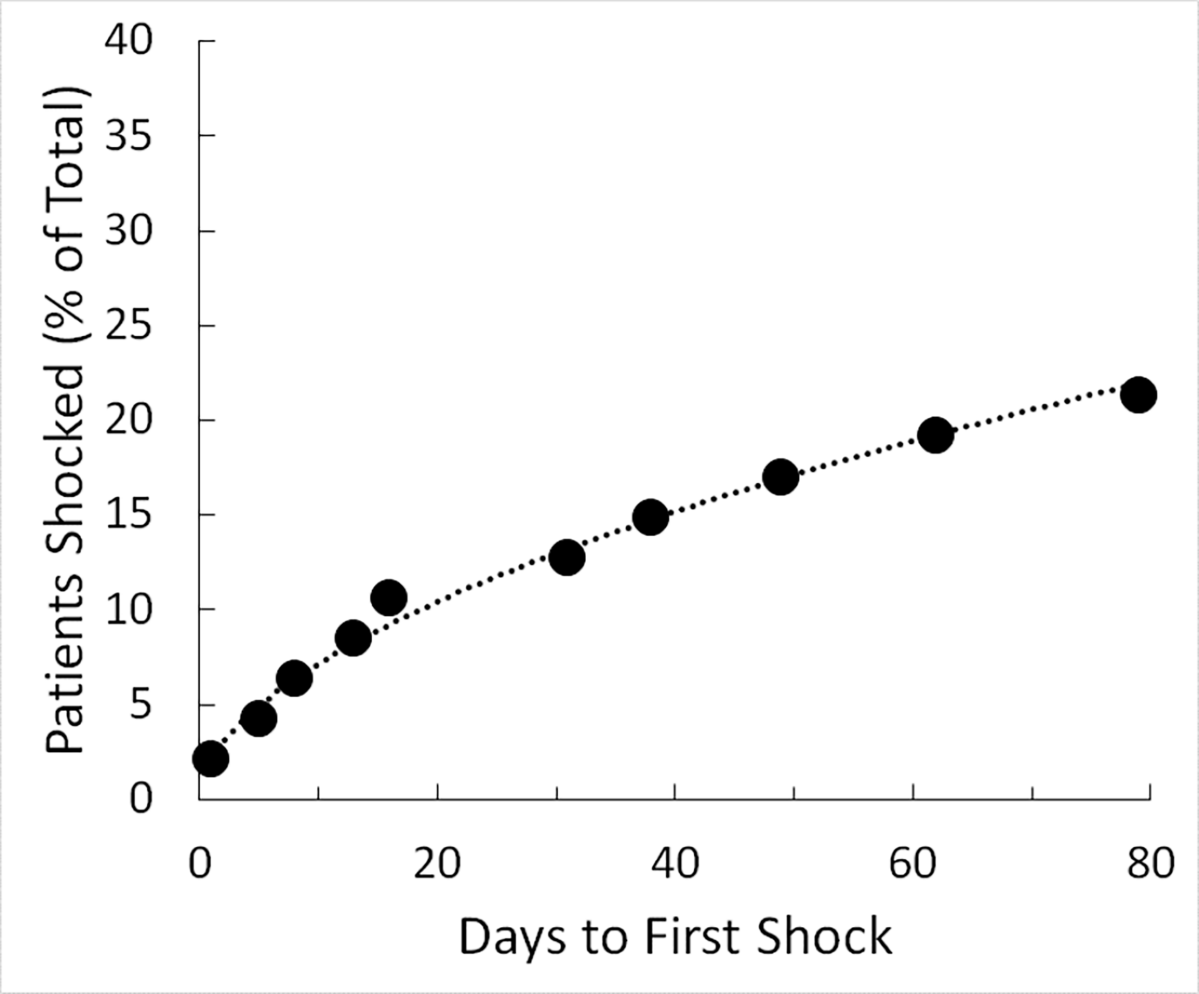
Days to appropriate shock. Days of WCD wear (x-axis) are plotted against the percentage of CS patients who had received an appropriate shock in response to VT or VF.

There were 10 patients who experienced 11 WCD treatments for VT/VF. Days of WCD wear prior to the initial VT/VF event ranged between 1 to 79 days with a median of 24 days (Fig 1). Conversion of the initial VT/VF event was 100% after the first shock in all patients. A second VT/VF occurred days later in 1 patient that required 4 shocks before converting.

**Fig 2:**
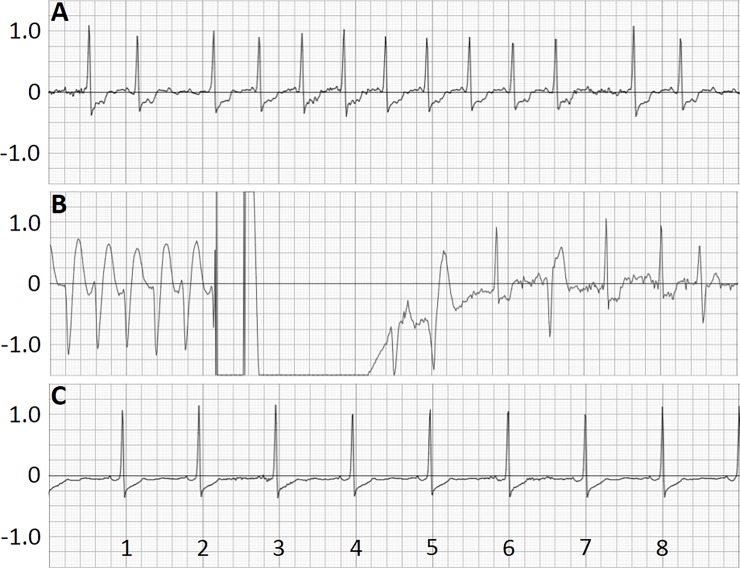
Electrocardiogram showing 9 seconds of data recorded by the WCD side-to-side channel electrodes. (A) Heart rhythm recorded by the WCD 4 days prior to VT. (B) Shock (150J) delivered by the WCD in response to sustained VT and recovery of sinus rhythm. (C) Heart rhythm recorded 7 days after delivery of shock. Recording speed (x-axis) is 25mm/second and amplitude scale (y-axis) is 1mv/20mm. Elapsed time (seconds) following the beginning of the recording are indicated.

The ECG rhythm, including the rhythm leading to delivery of shock, was captured by the WCD system. Examples of the ECG recordings are shown in Fig 2. At 4 days before shock the heart rhythm was atrial fibrillation with S-T depression (Fig 2A). Panel B contains the ECG encompassing the shock for VT and the resulting recovery of sinus rhythm along with accompanying ventricular ectopic beats. The ECG recording at 7 days after delivery of shock shown in Fig 2C, shows sinus rhythm with S-T depression.

Evaluation of the ECG records from shocked patients exposed 5 episodes of VT, 3 of VF and 3 of VT degenerating into VF were treated. Survival 24 hours after shock treatment was excellent with all patients surviving the VT/VF episode. Analysis of shock delivered to individual patients showed that over 6.2 patient-years of cumulative WCD wear there were 11 episodes of VT or VF. Shock treatments were delivered to 10 patients, with one patient shocked on day 5 and again on day 32.

#### Post shock death event

Among the shocked treated CS patients there was 1 death reported that occurred after WCD use was stopped. This 77 year old female had renal disease, congestive heart failure and a history of ventricular arrhythmia. The patient received shock treatment for 2 VT events. During the first VT event, conversion to stable heart rhythm occurred after the first shock treatment (Fig 2B). The second event occurred after an additional 28 days of WCD use but required 4 shocks for conversion of the VT. After the second VT event the patient was admitted to the hospital, WCD use was discontinued and death occurred in the hospital 12 days post admission.

## Discussion

The present study is the first to evaluate the efficacy of the WCD in a cohort of CS patients. The 46 patients identified in the WCD database exhibited 11 episodes of VT/VF occurring in 10 patients. Post shock survival was 100% and the WCD was effective in converting VT/VF. The median hours of daily WCD wear was 23.8 and 23.3 hours for shocked and not shocked patients. This is slightly greater than the 21.7 to 21.8 hours that have been reported for non-CS patients [17–20]. Moreover, the high rate of successful conversion and survival after arrhythmic episode provides a useful, although preliminary, study of management of SCD risk among this population.

At the end of the WCD use, ICD implantation was performed in 50% of patients. With respect to reassessment of LVEF after heart failure medication optimization and immunosuppression, there is no research to inform the timing of LVEF reassessment, and the authors of a consensus document suggest that this should be individualized and should occur at least 3 months after the intervention [6]. One may speculate that CS patients are at higher risk than patients with other structural heart diseases due to the sarcoid lesions/granuloma and a wide and spreading fibrosis in the myocardium; indeed cohorts of patients with CS and ICD have more frequent therapies from their ICDs than other groups [21]. In contrast, corticosteroids may be effective at reducing systemic inflammation and preventing organ damage, especially in the early phase of CS. Kandolin et al. recently reported a 10 year transplantation-free survival of 83% in 110 patients with CS and with immunosuppressive therapy [22]. However, the effect of corticosteroids on VT/VF is not consistent, therefore, a consensus paper on CS discussed the utility of WCD use in individual cases until improvement by corticosteroids can be determined [8]. The observed outcomes in our series are in line with previous publications and underscore the poor prognosis of patients with CS.

The diagnosis of CS for the patients included in this study was identified from the physician-provided clinical records authorizing the WCD prescription. The diagnosis of CS is sometimes difficult. Because the sensitivity of endomyocardial biopsy is low (<25%) [5], there is the need for non-invasive diagnostic methods, e.g. MRI and/or positron emission tomography. However, there is an increase in the detection of subclinical cardiac manifestation of sarcoidosis by these techniques. A nationwide study in Finland reported that the annual detection rate of CS has increased >20 fold over a 25 year period [22]. Whether the higher prevalence of CS is a reflection of improved diagnostic modalities or increased incidence of the disease itself needs further investigation. Another study showed that the presence of myocardial scar identified through late gadolinium enhancement by MRI was the best independent predictor of potentially lethal events [4]. There is disagreement in the literature as to the prognosis of clinically silent CS and the present study neither supports or rejects WCD use in CS patients with normal ejection fraction.

The retrospective study has several strengths and limitations. Strengths include the relatively large cohort of consecutive patients newly diagnosed with CS who were followed for a median of 33 days and for as long as 243 days, and the availability of physician generated patient history and physical examination indicating the presence of CS and cardiac co-morbidity with high risk of VT/VF. The cohort is one of the largest of CS patients and the first with CS and WCD use. Limitations include the paucity of data on the underlying sarcoidosis; data on lung function, blood gases, computed tomography (CT) findings and histology were not captured in the database. Additional limitations include the lack of systemic long-term follow up assessments, such as data on prescribed medications, and a control group of CS patients who did not receive the WCD.

### Conclusions

The retrospective study describes a population of patients with high risk of SCD persisting for at least the first 79 days of use. The relatively high shock frequency and survival for 24 hours post shock treatment underscore the efficacy provided by rapid access to defibrillation. The WCD can prevent SCD in sarcoidosis patients with known, or suspected, cardiac involvement until optimized long term treatment strategies can be attained. Due to the need for ongoing immunosuppression therapy and the potential for some patients to improve, not all patients should be immediately implanted with an ICD. Application of the WCD to the management of CS patients has the potential to reduce SCD prior to the decision that permanent arrhythmia risk exists and ICD implantation is required.

## Supporting information

S1 Table Supporting information file with raw data underlying the study. (XLSX)
